# Good Samaritan Eye and Ear Infirmary

**Published:** 1887-02

**Authors:** 


### *GOOD SAMARITAN EYE AND EAR INFIRMARY.*

The annual meeting of the trustees of this institution was held last month for the election of officers and the reception of the surgeon's report. The regular officers of the board were elected as follows: President, T. V. Dickinson; vice president, Albert Jones; secretary, W. H. Slocum; treasurer, A. A. Hubbell. Dr. R. L. Banta of this city and Dr. John O. Roe of Rochester were added to the consulting staff, and Dr. F. H. Potter was appointed surgeon to the throat and nose department, which was made separate from the eye and ear department. Heretofore, the entire surgical management has been under Dr. A. A. Hubbell, who has most faithfully given daily attention to those who have applied for treatment, but on his own recommendation the service was divided into two departments. This infirmary has been in operation since August 1, 1882, and has been doing a good work in administering to the *worthy poor*, and these alone. Patients are received for free treatment, but only on giving evidence of their inability to pay for services, or on the recommendation of physicians, thus avoiding that abuse of medical charity which is too frequently a just cause of complaint. With the support that the infirmary receives from the medical profession, and with the new accessions to its medical staff, its increased usefulness is assured for the future.

Dr. Potter, the surgeon of the throat department, has just returned after a prolonged course of instruction under Professor Shurley, of Detroit. He has relinquished general practice, and will limit it to this specialty, in which he is well qualified.

